# Inhibition of tumor promoting signals by activation of SSTR2 and opioid receptors in human breast cancer cells

**DOI:** 10.1186/1475-2867-13-93

**Published:** 2013-09-23

**Authors:** Geetanjali Kharmate, Padmesh S Rajput, Yu-Chen Lin, Ujendra Kumar

**Affiliations:** 1 Faculty of Pharmaceutical Sciences, The University of British Columbia, Vancouver, BC V6T1Z3, Canada

**Keywords:** Breast cancer, Mitogen activated protein kinases, Opioid receptors and somatostatin receptor-2

## Abstract

**Background:**

Somatostatin receptors (SSTRs) and opioid receptors (ORs) belong to the superfamily of G-protein coupled receptors and function as negative regulators of cell proliferation in breast cancer. In the present study, we determined the changes in SSTR subtype 2 (SSTR2) and μ, δ and κ-ORs expression, signaling cascades and apoptosis in three different breast cancer cells namely MCF-7, MDA-MB231 and T47D.

**Methods:**

Immunocytochemistry and western blot analysis were employed to study the colocalization and changes in MAPKs (ERK1/2 and p38), cell survival pathway (PI3K/AKT) and tumor suppressor proteins (PTEN and p53) in breast cancer cell lines. The nature of cell death upon activation of SSTR2 or OR was analysed using flow cytometry analysis.

**Results:**

The activation of SSTR2 and ORs modulate MAPKs (ERK1/2 and p38) in cell dependent and possibly estrogen receptor (ER) dependent manner. The activation of tumor suppressor proteins phosphatase and tensin homolog (PTEN) and p53 antagonized the PI3K/AKT cell survival pathway. Flow cytometry analyses reveal increased necrosis as opposed to apoptosis in MCF-7 and T47D cells when compared to ER negative MDA-MB231 cells. Furthermore, receptor and agonist dependent expression of ORs in SSTR2 immunoprecipitate suggest that SSTR2 and ORs might interact as heterodimers and inhibit epidermal growth factor receptor phosphorylation.

**Conclusion:**

Taken together, findings indicate a new role for SSTR2/ORs in modulation of signaling pathways involved in cancer progression and provide novel therapeutic approaches in breast cancer treatment.

## Background

Somatostatin (SST) is a multifunctional growth hormone inhibitory neuropeptide, regulating different arrays of functions in the brain, endocrine and exocrine tissues. One of the prominent functions of SST is the negative regulation of cell proliferation in normal as well as pathological conditions including pituitary, pancreatic and breast tumors [1-5]. The anti-proliferative effects of SST occur indirectly through the inhibition of growth factors such as insulin growth factor-1 and epidermal growth factor and angiogenesis [6-9]. The direct anti-proliferative effect of SST is by binding to seven transmembrane G-protein coupled receptors (GPCRs) namely somatostatin receptors 1–5 (SSTR1-5). This direct effect of SST is either cytostatic (cell cycle arrest) or cytotoxic (apoptosis) [2,10-12]. SSTR1-5 subtypes are variably expressed in various human tumors including breast cancer tissues and cells [4,12,13]. We have previously shown a significant correlation between mRNA and protein expression of SSTRs with histological tumor markers as well as with expression levels of estrogen receptors (ER) and progesterone receptors [4]. These results indicate that the presence of hormone receptors might play crucial role on SSTR effectiveness in breast cancer.

SST and SSTRs are highly expressed in breast cancer cells as well as autopsied breast tissue. However, SSTR2 is the prominent receptor subtype expressed ubiquitously and abundantly in breast tumor tissues and cancer cells. Vikic-Topic et al., described that SSTR2 transcript is predominantly expressed in all breast tissue samples and followed by SSTR1, SSTR3 and SSTR4 [14]. Additionally, Pfeiffer et al., reported that SSTR2 and SSTR5 as the predominant subtypes expressed in primary breast tumors [15]. Moreover, MCF-7 cells with over-expression of SSTR2 display diminished rate of cell proliferation [16]. SSTR2 exerts its anti-proliferative effect by either activating or suppressing various signal transduction pathways including mitogen activated protein kinases (MAPK), phosphatidylinositol-3-protein kinase (PI3K)/AKT, phosphotyrosine phosphatases such as PTP1 and PTP2 [17-21]. The activation of multiple signaling pathways consequently leads to the induction of cell cycle arrest via activation of cyclin dependent kinase inhibitor (p27^Kip1^) as well as apoptosis. SSTRs positive tumors are less malignant with higher survival rate whereas the lack of SSTRs expression has also been associated with poorly differentiated and invasive tumor [22]. This could partly be attributed to the over-expression of epidermal growth factor receptors (EGFRs) in breast cancer that are associated with poor prognosis and patient survival rate [13,23,24]. Importantly, in MCF-7 cells, over-expression of SSTR2 resulted in suppression of EGFR expression [16]. Further in support, our recent studies have also described attenuation of EGFR phosphorylation and suppression of tumor promoting signals in breast cancer cells as well as in human embryonic kidney (HEK)-293 cells transfected with SSTR1 or SSTR5 [13,20,21].

Like SSTRs, opioid receptor (ORs), namely μ, δ and κ are also the members of GPCR family. SSTRs and ORs share >40% structural homology and are well expressed in various breast cancer cells as well as in solid breast tumor tissues [25,26]. ORs are well characterized for their analgesic role and like SSTRs have recently been reported as negative regulators of cell growth in various tumors including, prostrate, lung, kidneys and breast cancer [27-29]. It has been previously shown that >50% of invasive ductal carcinomas are positive for opioid peptide like immunoreactivity [30]. Furthermore, human adrenocarcinoma and breast cancer cell lines exhibited the binding site for opioids. The opioid agonist [_D_-Ala^2^, _D_-Leu^2^] enkephalin (DADLE), displayed inhibition of cell proliferation in a concentration dependent manner and was reversed in the presence of antagonist naloxone [29,31,32].

SSTR and OR subtypes constitute functional heteromeric complexes within same sub-family and other GPCRs and modulate receptor trafficking and signaling properties [25,33-37]. SSTR5 and dopamine receptor 2 (D2R) heterodimerization synergistically control the hyper-secretion of growth hormone and prolactin in pituitary adenomas [38,39]. These observations have led to the application of new chimeric molecules of D2R and SSTR5 for the treatment of pituitary tumor acromegaly [1,38-40]. Furthermore, SSTR2 interfere with PI3K signaling via disruption of the SSTR2/p85 subunit complex consequently inhibiting the cell proliferation and tumor growth [17]. HEK-293 cells co-transfected with SSTR2 and μOR constituted stable heterodimers thereby regulating the receptor phosphorylation, internalization and desensitization [15]. Whether SSTR2 functionally interacts with ORs in breast cancer cells expressing these receptors endogenously and function in similar manner as described in heterologous system is largely elusive. We hypothesize that the simultaneous activation of SSTR2 and ORs may exert pronounced anti-proliferative effect via changes in signaling pathways in breast cancer cells. Multiple studies have documented that estrogen upregulated the expression of SSTR2 mRNA and protein via ER in T47D and ZR75-1 (ER + ve) breast cancer cells. These findings may anticipate the role of SSTR2 in ER responsiveness of breast cancer [41-43]. However, SSTR2 and OR subtypes mediated effect on signaling pathways in part are dependent on the presence of ER in breast cancer cells is still elusive. In the present study, we focus to determine the expression of SSTR2 and ORs and the changes in receptor expression and signaling pathways upon treatment with receptor specific agonist in human breast cancer cell lines; MCF-7 (ER + ve), MDA-MB231 (ER-ve), and T47D (ER + ve).

## Materials and methods

### Chemicals and reagents

SSTR2 specific non-peptide agonist L-779,976 was provided by Dr. Rohrer from Merck & Co. Specific agonists for μOR (DAMGO), δOR ([D-Ala^2^]- Deltorphin II) and κOR (±)-U-50488 hydrochloride) were purchased from Tocris Biosciences (Ellisville, MO). Polyclonal rabbit anti-SSTR2 antibody was developed in our laboratory [44,45]. The antibodies against ORs (μ, δ and κ), phosphorylated and total-EGFR were obtained from Santa Cruz Biotechnology (Santa Cruz, CA). Rabbit polyclonal antibodies against phosphorylated and total-ERK1/2, p38, PI3K, AKT, PTEN and p53 were purchased from Cell Signaling Technology (Mississauga, ON). Goat anti-rabbit or donkey anti-goat Alexa Fluor-488 and Alexa Fluor-594 were purchased from Invitrogen (Burlington, ON). Annexin-APC V was obtained from BD Biosciences (Mississauga, ON). All experiments were performed in compliance with Office of Research and Biosafety Committee guidelines at the University of British Columbia.

### Cell lines and culture

Human breast cancer cell lines, MCF-7 and T47D (ER + ve) were maintained in RPMI 1640 medium supplemented with 10% (v/v) fetal bovine serum (FBS) and 1% antibiotic (penicillin/streptomycin) at 37°C, 5% CO_2_ as previously described [4,13]. MDA-MB231 (ER-ve) cells were maintained in Leibovitz’s L-15 medium supplemented with 10% (v/v) FBS and 1% antibiotic (penicillin/streptomycin) at 37°C in a CO_2_ free atmosphere.

### Indirect immunofluorescence immunocytochemistry

Breast cancer cells were processed for indirect immunofluorescence immunocytochemistry as described earlier [13,21]. Briefly, cells were washed and fixed with 4% paraformaldehyde and followed by treatment with Triton X-100 for 10 min at room temperature. The cells were incubated with anti-goat μ, δ, and κ -OR (1:300) primary antibodies overnight at 4°C and followed by incubation in goat-anti-rabbit Alexa 488 (1:700) conjugated secondary antibodies for 1 h at room temperature for final color development. The cells were viewed and photographed on a Leica DMLB microscope attached to a Retiga 2000R camera. The specificity of immunoreactivity was determined in absence of primary antibodies or in presence of pre-immune serum as described earlier [45].

### Western blot analyses

Membrane extracts as well as whole cell lysate prepared from control and treated cells were fractionated on SDS-PAGE as described previously [13,21]. To determine the receptor expression in membrane extracts, blots were incubated with primary antibodies against SSTR2 (1:400) and μ, δ and κ-OR (1:500). The status of signaling molecules were examined by incubating the immunoblots with antibodies against phosphorylated and/or total extracellular regulated protein kinase 1/2 (ERK1/2), p38, PI3K, AKT, PTEN and p53 (1:1000). Membrane was incubated with peroxidase conjugated secondary antibodies respectively. The bands were detected using chemiluminescence in accordance to the manufacturer’s instructions (Amersham Biosciences). Images were captured using the Alpha Innotech FluorChem 8800 gel box imager and FluorChem software was used to quantify the blots. Tubulin was used as the loading control.

### Co-Immunoprecipitation (Co-IP)

Breast cancer cells were treated with specific agonists for SSTR2, L-779,976 (10 nM) and μOR (DAMGO; 1 μM), δOR (Deltorphin-II; 1 μM), or κOR (U50488HCl; 1 μM) alone or in combination for 15 min at 37°C. 200 μg of total membrane protein was solubilized in 1 ml of radio-immune precipitation assay (RIPA) buffer (150 mM NaCl, 50 mM Tris–HCl, 1% Nonidet P-40, 0.1% SDS, 0.5% sodium deoxycholate, pH 8.0) for 1 h at 4°C as described earlier [20,21]. Samples were incubated with anti-SSTR2 antibody (1:200) for immunoprecipitation and purified with protein A/G-agarose beads overnight at 4°C. Purified proteins were subjected to 7% SDS-PAGE and probed for the expression of ORS using anti-μ, δ and κ-OR antibodies (1:500) respectively as described previously [20,21].

### Flow cytometry analysis for apoptosis/necrosis

Cells were harvested and treated with L-779,976, DAMGO, Deltorphin-II, or U50488HCl alone and/or in combination for 30 min at 37°C. Annexin V-APC staining was performed according to the manufacturer’s protocol (BD Biosciences). Cells were washed twice with cold PBS and resuspended in 1X binding buffer (0.1 M Hepes, pH 7.4, 1.4 M NaCl, 25 mM CaCl_2_) at a cell density of 1 × 10^5^cells/ml. Annexin V-APC and propidium iodide were added to the cell suspension for 15 min. Prior to flow cytometry, 400 μl of 1X binding buffer was added and cells were analyzed using FACS Calibur flow cytometer (Becton Dickinson, San Jose, CA). A minimum of 10,000 events were recorded for each sample. Cells positive for Annexin V-APC were identified as apoptotic whereas cells positive for both Annexin V-APC and propidium iodide were characterized as necrotic.

### Statistical analysis

The changes in the expression of proteins were quantified using ANOVA and *post hoc* Dunnett’s or Bonferroni’s tests. Statistical analysis was performed using GraphPad Prism 4.0 to determine the significant changes. Significant statistical differences were taken at *p < 0.05. Results are presented as mean ± SEM from three independent experiments (*n = 3*).

## Results

### Comparative distribution of SSTR2 and ORs in MCF-7, MDA-MB231 and T47D cells

**Figure 1:**
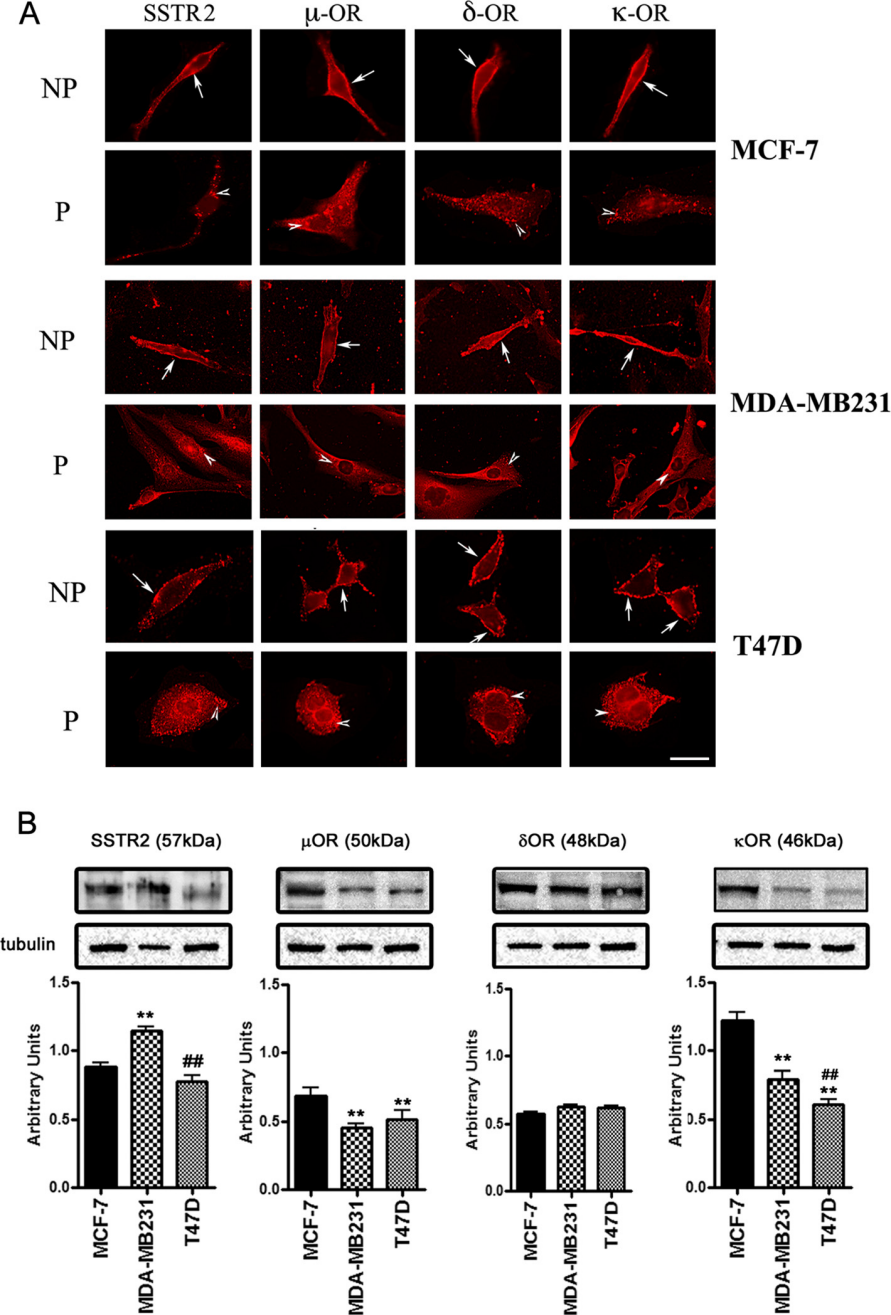
**Differential expression of SSTR2 and μ, δ, κ-ORs in human breast cancer cells. (A)** Indirect immunofluorescence staining showing membrane (non-permeabilized, **NP**) and intracellular (permeabilized, **P**) expression of SSTR2 and μ, δ, κ-ORs in breast cancer cells. In the representative panels receptor expression at the cell surface is indicated by arrows whereas arrowheads indicate intracellular expression. **(B)** Cell membrane extracts obtained from MCF-7, MDA-MB231 and T47D breast cancer cells were subjected to Western blot analysis and probed with specific primary antibodies to detect the receptor expression. The immunoblots show differential expression of SSTR2 (~57 kDa) and μ (~50 kDa), δ (~48 kDa), κ (~46 kDa)-ORs in a cell specific manner. Histogram represents densitometric analysis of the receptor expression normalized by using tubulin as loading control. Results are expressed as mean ± SEM (*n = 3*). **p < 0.01, MCF-7 vs. MDA-MB231 or T47D cells; **##*p < 0.01****;* MDA-MB231 vs. T47D. Scale bar 10 μm.

SSTR2 and ORs expression at the cellular levels and in membrane fractions was accomplished by immunofluorescence immunocytochemistry and western blot analysis respectively. MCF-7 cells, displayed strong membrane expression of SSTR2 and μ, δ, κ-ORs whereas intracellular expression of SSTR2 was weak than the ORs (Figure 1A). In MDA-MB231 cells, SSTR2 and μ, δ and κ-ORs like immunoreactivity was observed at the cell surface with a dominant expression of δ and κOR, whereas, the receptors expression in the cytoplasmic compartment was comparable (Figure 1A). In contrast, T47D cells displayed strong expression of SSTR2 and μ, δ, κ-ORs at the cell surface as well as intracellularly (Figure 1A).

To support the cellular distribution by immunocytochemistry receptor like immunoreactivity was also confirmed using Western blot analysis. As shown in Figure 1B, SSTR2 was well expressed at the expected molecular size of ~57 kDa with relatively higher expression in MDA-MB231 cells in comparison to MCF-7 and T47D. The expression level of SSTR2 was comparatively less in T47D cells than MCF-7 and MDA-MB231 cells. In contrast, μOR (~50 kDa) and κOR (~46 kDa) were well expressed in MCF-7 cells. Conversely, the expression of κOR in membrane extract prepared from MDA-MB231 and T47D cells was relatively weak (Figure 1B). The expression of δOR (~48 kDa) was comparable in all three cell lines. These observations indicate cells-specific expression of SSTR2 and ORs.

### SSTR2 and ORs modulate MAPKs in a cell-specific manner

**Figure 2:**
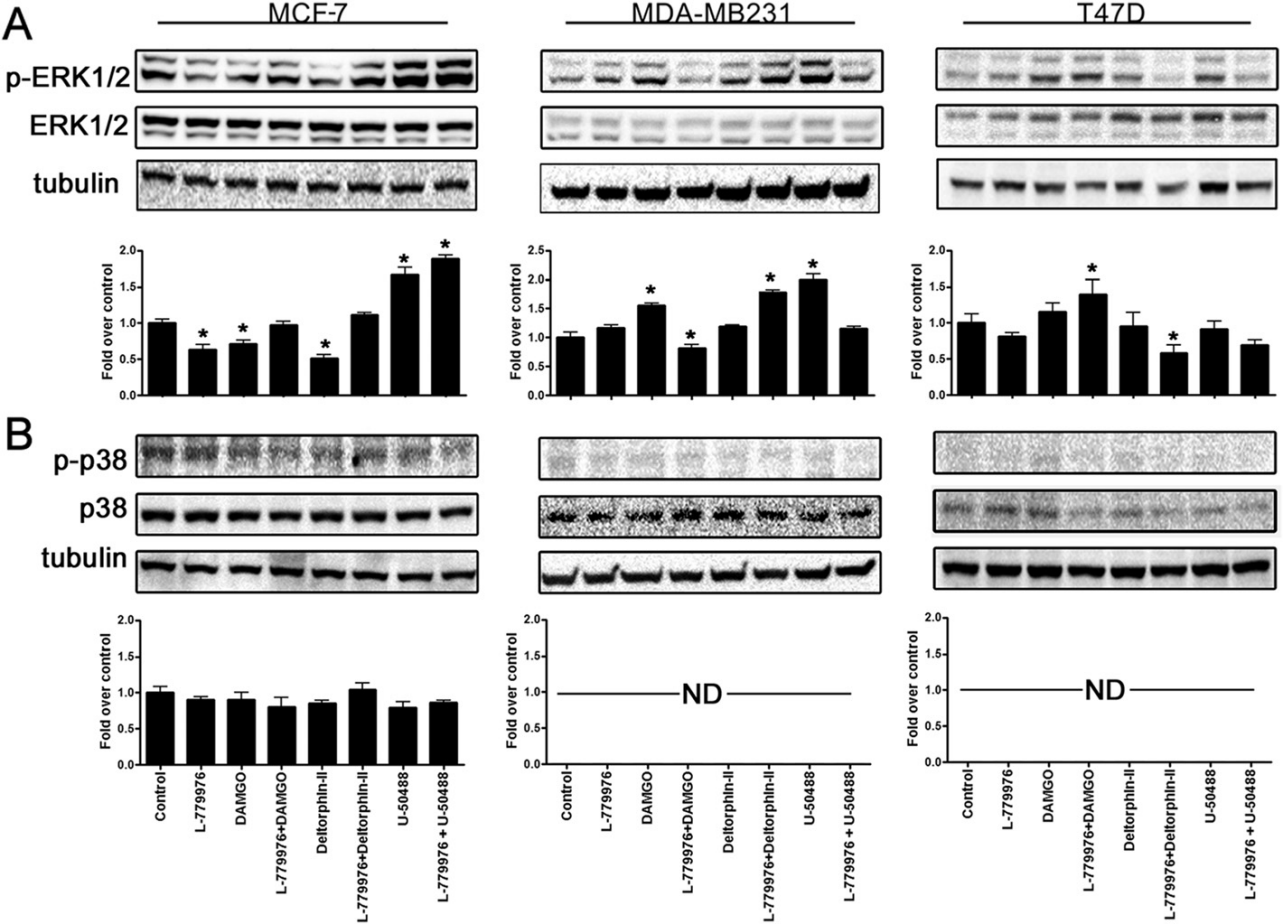
**MAPKs (ERK1/2 and p38) are modulated in a receptor and cell-specific manner.** Whole cell lysates obtained from MCF-7, MDA-MB231 and T47D cells following treatment with SSTR2 and ORs agonists alone and/or in combination were subjected to western blot analysis and probed for phospho-and total ERK1/2 and p38 (1:1000). **(A)** Immunoblots illustrating agonist mediated changes in phosphorylated ERK1/2 in cell-specific manner.** (B)** Immunoblots displaying changes in the phosphorylation of p38 upon specific agonist treatments in breast cancer cells. SSTR2 and ORs activation inhibited p38 phosphorylation upon indicated treatment. Histograms depict changes in the expression levels of ERK1/2 using densitometric analysis. The data presented here is a representation mean ± SEM of three independent experiments. Significant difference was considered at *p < 0.05 vs. control.

We next determined whether receptor activation regulate MAPKs (ERK1/2 and p38) in breast cancer cells. In MCF-7 cells, L-779,976, DAMGO and Deltorphin-II alone inhibit the phosphorylation of ERK1/2 (p-ERK1/2) (Figure 2A). Furthermore, L-779,976 in the presence of DAMGO or Deltorphin-II displayed p-ERK1/2 comparable to control. In contrast, U50488HCL, alone or in combination with L-779,976 significantly elevated p-ERK1/2 in MCF-7 cells (Figure 2A). In MDA-MB231 cells, L-779,976 alone had no significant effect on p-ERK1/2. DAMGO alone induced p-ERK1/2 whereas in combined treatment with L-779,976 decreased p-ERK1/2. In contrast, Deltorphin-II alone had no significant effect on p-ERK1/2 whereas in combination with L-779,976 enhanced the levels of p-ERK1/2 in MDA-MB231 cells (Figure 2A). Furthermore, in MDA-MB231 cells the activation of κOR enhanced p-ERK1/2 which was significantly decreased to the control level upon combined treatment with SSTR2 agonist L-779,976. In T47D cells, L-779,976 maintained p-ERK1/2 comparable to control. The activation of μOR displayed comparable p-ERK1/2 but significantly increased in combination with L-779,976. The status of p-ERK1/2 was not changed upon activation of δOR alone whereas expression level was diminished significantly in presence of L-779,976 and Deltorphin-II. Activation of κOR alone had no effect on p-ERK1/2 however simultaneous activation of SSTR2/κOR inhibited p-ERK1/2 when compared to control (Figure 2B).

In addition to ERK1/2, in tumor cells, p38 is a crucial mediator of apoptosis, cell-cycle arrest, cell differentiation and tumor suppression [46,47]. The pro- and/or anti-apoptotic role of p38 is attributed to the cell-type and stimuli. Of note, p38 phosphorylation remained comparable to control in MCF-7 cells upon treatments with SSTR2 and ORs agonists alone or in combination (Figure 2B). Unlike MCF-7 cells, the p-p38 was not detected in MDA-MB231 and T47D cells across all indicated treatments. Collectively, these data suggest that basal expression of p-p38 is relatively higher in MCF-7 cell in comparison to MDA-MB231 or T47D cells without any discernible changes upon SSTR2 and ORs activation.

### SSTR2 and ORs maintained basal activation of PI3K/AKT

**Figure 3:**
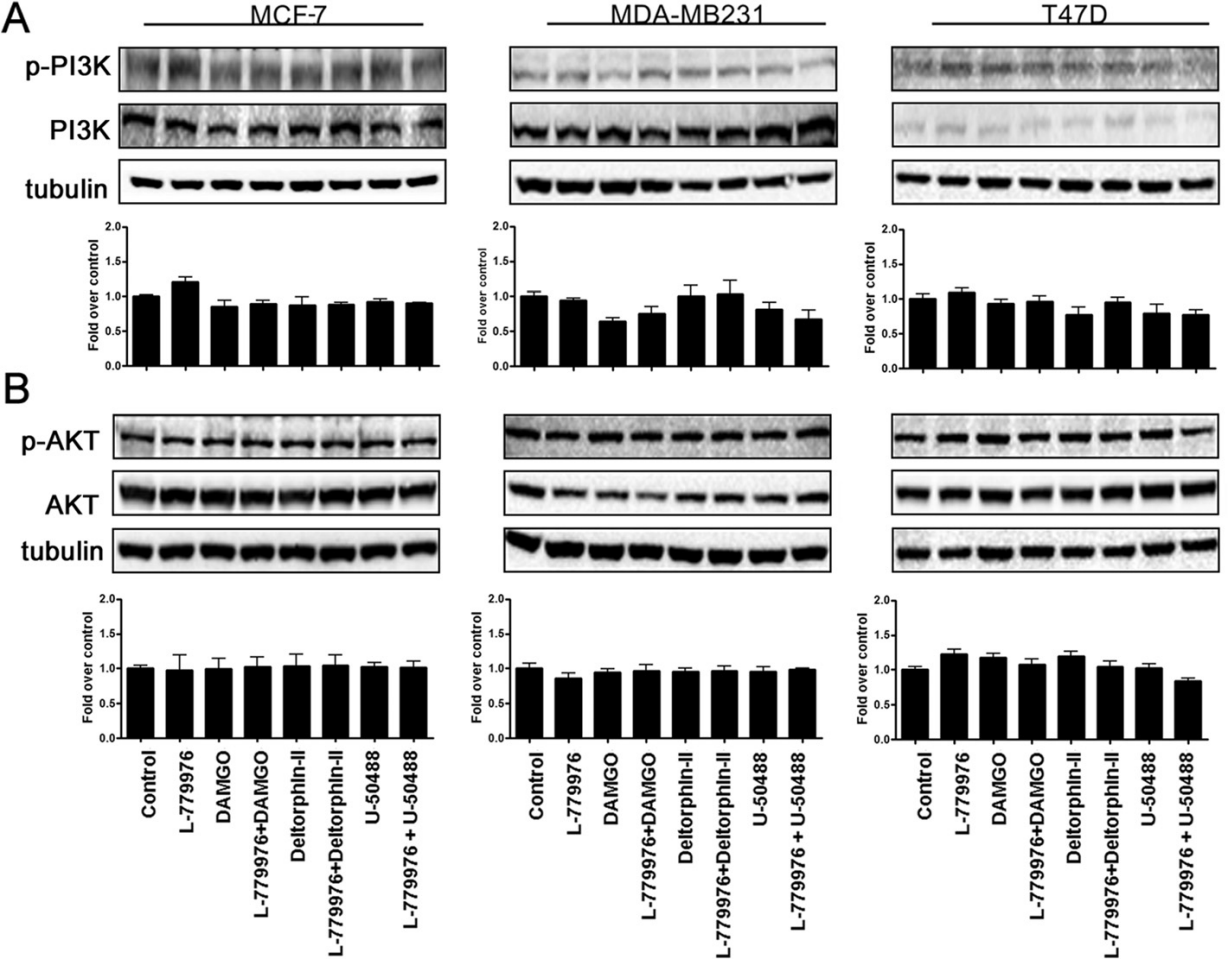
**Activation of SSTR2 and ORs modulate PI3K/AKT cell survival pathway.** Representative western blots illustrating the changes in the expression of PI3K **(A)** and AKT **(B)** in MCF-7, MDA-MB231 and T47D cells. Activation of SSTR2 and ORs individually or simultaneously exhibited no discernible changes in phosphorylation of PI3K and AKT, thus maintaining the status of PI3K/AKT at control levels. Bar graphs represent the densitometric analysis of PI3K/AKT expression in different tumor cells. The data presented are a representation of triplicate experiments.

The aberrant activation and/or mutations in PI3K are associated with tumor growth and failure of hormonal therapy [48-50]. Accordingly, we next determined the status of phosphorylated PI3K/AKT in control and cells treated with SSTR2 and ORs specific agonists. No significant changes in the status of PI3K phosphorylation was observed in all cell lines upon indicated treatments, albeit lower levels of p-PI3K when compared to control (Figure 3A). Furthermore, irrespective to treatment and cell lines the status of AKT phosphorylation was without any significant changes across all treatments in all three breast cancer cells as indicated (Figure 3A).

### SSTR2 and ORs modulate tumor suppressor proteins PTEN and p53 in cell dependent manner

**Figure 4:**
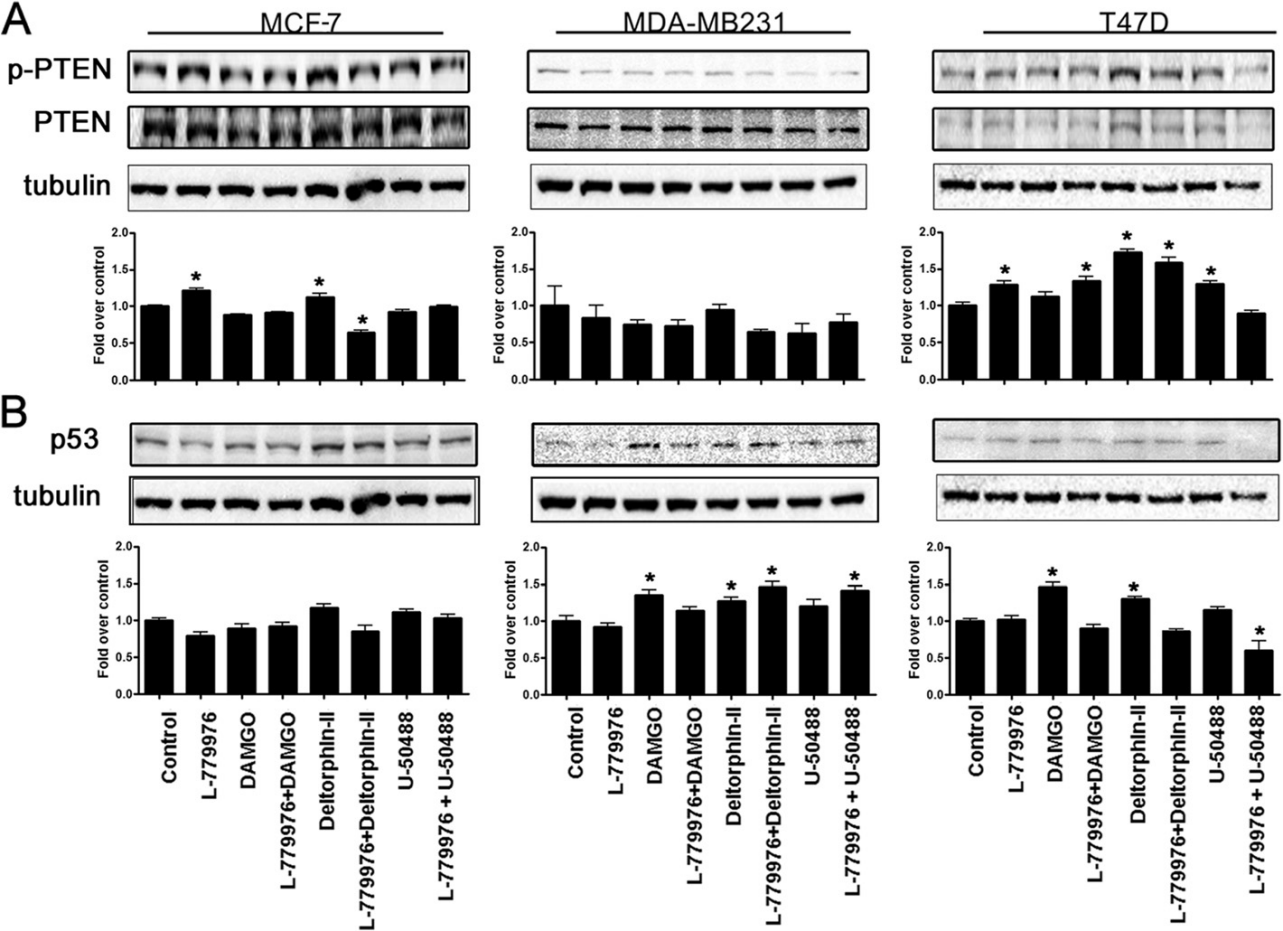
**Tumor suppressor proteins PTEN and p53 are modulated in cell and receptor specific manner in tumor cells.** Representative immunoblots showing the effect of SSTR2 or ORs activation on the expression of PTEN **(A)** and p53 **(B)** in MCF-7, MDA-MB231 and T47D cells respectively. **(A)** The expression of PTEN is enhanced in MCF-7 as well as T47D cells; however the MDA-MB231 cells displayed no discernible changes in the levels of PTEN. **(B)** The expression of p53 was in a receptor and cell-specific manner. Note the comparable changes in PTEN and p53 expression in MCF-7 and T47D cells. Histograms illustrate densitometric analysis of PTEN and p53 levels using tubulin as the loading control. The results presented here are representation of mean ± SEM, *n = 3*. Significant difference was considered at *p < 0.05 vs. control.

Tumor suppressor proteins, PTEN and p53 serve as negative regulators of cell proliferation in breast cancer and mutations in PTEN and p53 are often associated with the activation of AKT cell survival pathway [49]. In MCF-7 cells, L-779,976 alone significantly enhanced the phosphorylation of PTEN whereas DAMGO alone or in combination with L-779,976 had no effect on p-PTEN (Figure 4A). Upon treatment with Deltorphin-II alone MCF-7 cells displayed elevated p-PTEN however co-activation of SSTR2 and δOR resulted in significant inhibition of p-PTEN comparable to control. Furthermore, in presence of U50488HCl alone or in combination with L-779,976 the status of p-PTEN was without any significant effect in MCF-7 cells (Figure 4A). Conversely, in MDA-MB231 cells there was no discernible change in p-PTEN across all the treatments as indicated. In T47D cells, L-779,976 alone and in combination with DAMGO significantly increased p-PTEN whereas DAMGO alone enhanced PTEN phosphorylation insignificantly different than the control. Furthermore, Deltorphin-II alone and in combination with L-779,976 displayed significant increase in the p-PTEN. In T47D cells, κOR agonist resulted in significant activation of PTEN whereas such effect was not significantly different from control upon synergistic activation of SSTR2 and κOR (Figure 4A).

As shown in Figure 4A, MCF-7 displayed no discernible changes in the expression of p53 across all the treatments. In contrast, in MDA-MB231 cells, L-779,976 alone was without any effect on p53 expression. DAMGO alone enhanced the expression of p53 significantly whereas in combination with L-779,976, p53 level was similar to the control. Deltorphin-II alone and in combination with L-799,796 significantly elevated the levels of p53. Furthermore, p53 expression was unchanged upon activation of κOR alone whereas the combined treatment displayed significant increase in p53 expression in MDA-MB231 cells (Figure 4B). In T47D cells, L-779,976 alone and in combination with either DAMGO or Deltorphin-II exhibited no change in p53 expression. In contrast, DAMGO and Deltorphin-II alone significantly enhanced the p53 expression in comparison to the control. U50488HCl alone did not affect p53 expression however, in combination with L-779,976, the p53 levels significantly decreased (Figure 4B). Taken together these observations suggest that SSTR2 and ORs upregulated the PTEN and p53 expression in a receptor and cell dependent manner.

### SSTR2 and ORs induced early apoptosis and predominant cytostatic effect in cell dependent manner

**Figure 5:**
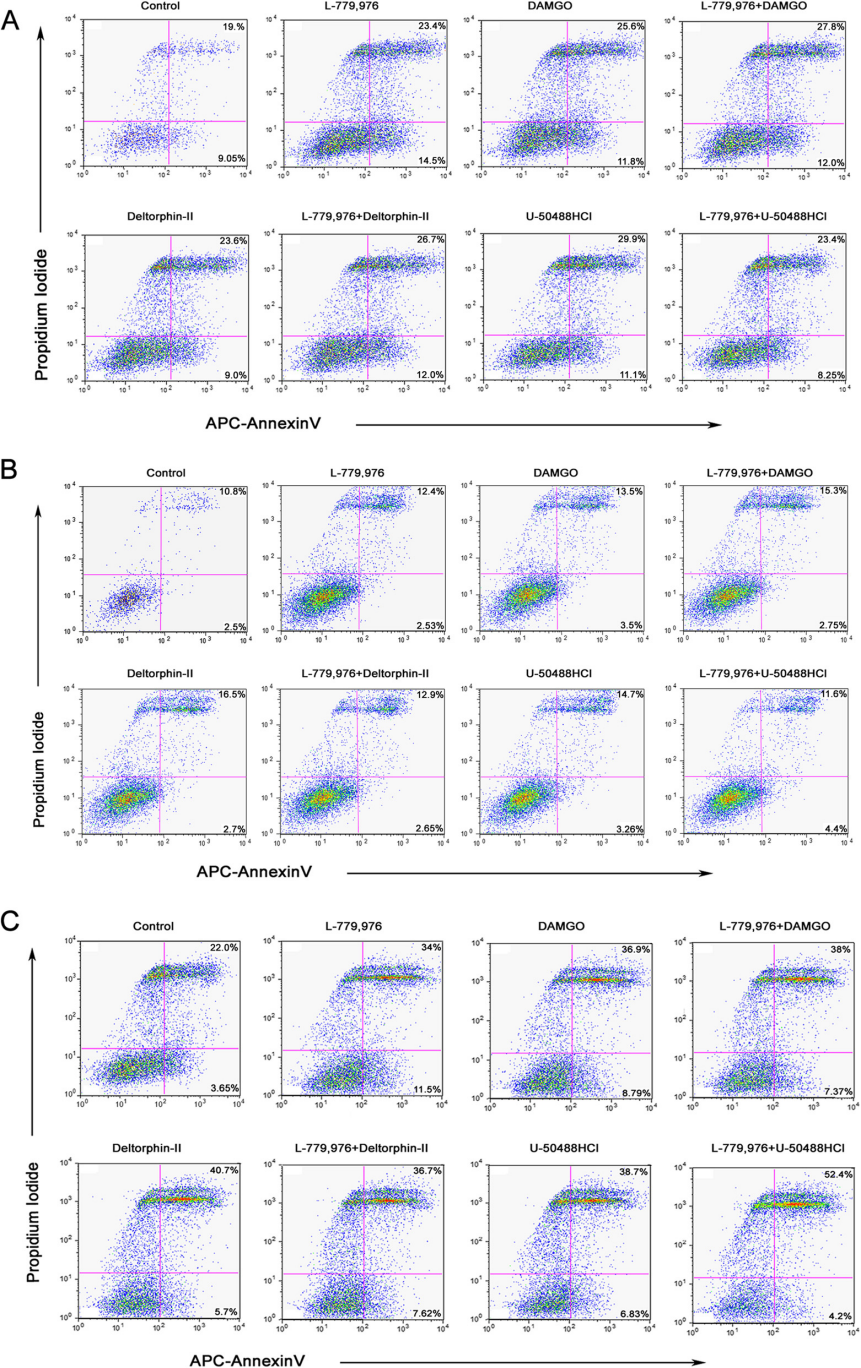
**Representative flow cytometry analysis displaying apoptosis and necrosis after exposure to SSTR2 and OR agonist in breast cancer cells.** Cells were harvested and treated with SSTR2 or ORs specific agonists alone and in combination for 30 min at 37°C as described in Material and Method section. The percentage of apoptosis (lower right quadrant) and necrosis (upper right quadrant) was evaluated in MCF-7 **(A)**, MDA-MB231 **(B)** and T47D **(C)** cells upon treatment as indicated. Flow cytometry profile illustrating APC-Annexin V staining on X-axis whereas PI staining on Y-axis. A minimum of 10,000 events were recorded for each sample.

The anti-proliferative effect of SSTR2 is mediated via two different mechanisms; cytostatic and cytotoxic. Accordingly, applying flow cytometry, we investigated the cellular response upon receptor specific agonist treatments. The results indicated apoptotic (9.05%) and necrotic (19%) cells in control MCF-7 cells whereas upon single treatment with L-779,976 number of the cells displaying apoptosis and necrosis was enhanced (14.5% and 23.4% respectively) (Figure 5A). In presence of DAMGO alone or with L-779,976 cells displayed higher extent of necrosis (25% and 27.8% respectively) without any significant changes in apoptosis (11.8% and 12%). Furthermore, the number of cells displaying apoptosis and necrosis was enhanced upon combined treatment with L-779,976 and Deltorphin-II (12% and 26.7%) than Deltorphin-II alone (9 and 23.6%, respectively). In contrast, the activation of κOR with U50488HCl alone caused higher necrosis (29.9%), but was decreased to 23.4% upon combined treatment with L-779,976 whereas cells entering apoptosis was limited to ~10% (Figure 5A).

As illustrated in Figure 5B, MDA-MB231 cells upon treatments with SSTR2 or μ, δ and κ-ORs agonists alone or in combination displayed <4% of apoptotic cell population. Conversely, cells undergoing necrosis increased (12.4%) upon treatment with L-779,976 alone in comparison to the control (10.8%). DAMGO alone enhanced the necrotic cells (13.5%) whereas upon combined treatment with L-779,976, the number of cells going through necrosis were enhanced to 15.3% in MDA-MB231 cells (Figure 5B). Deltorphin-II alone enhanced the necrosis to 16%; however, combined treatment with L-779,976 decreased necrosis to 12.9%, similar to that seen with L-779,976 alone (12.4%). Similarly, treatment with U50488HCl led to 14.7% necrosis and was decreased to 11.6% upon combined treatment with L-779,976 (Figure 5B).

In basal condition T47D cells number of the cells going through apoptosis and necrosis cells were 3.65% and 22% respectively (Figure 5C). The treatment with L-779,976 enhanced the apoptosis (11.5%), however necrosis was elevated (34%) when compared to the control. Interestingly, upon treatments with DAMGO, alone or in combination with L-779,976, the population of necrotic cells (36.9% and 38%) was enhanced significantly as compared to the early apoptotic cells (8.79% and 7.37%). Deltorphin-II alone or in combination with L-779,976 displayed higher necrosis (40.7% and 36.7%) than apoptosis (5.7% and 7.62%) (Figure 5C). Of note, combined treatment of U50488HCl and L-779,976 markedly increased the necrosis (52.4%) whereas exhibited only 4.2% apoptosis (Figure 5C). These data indicate that ER + ve cells (MCF-7 and T47D) are more susceptible to necrosis upon simultaneous activation of SSTR2 and ORs than ER-ve cells (MDA-MB231).

### Expression of ORs in SSTR2 immunoprecipitate is cell and receptor-specific in tumor cells

**Figure 6:**
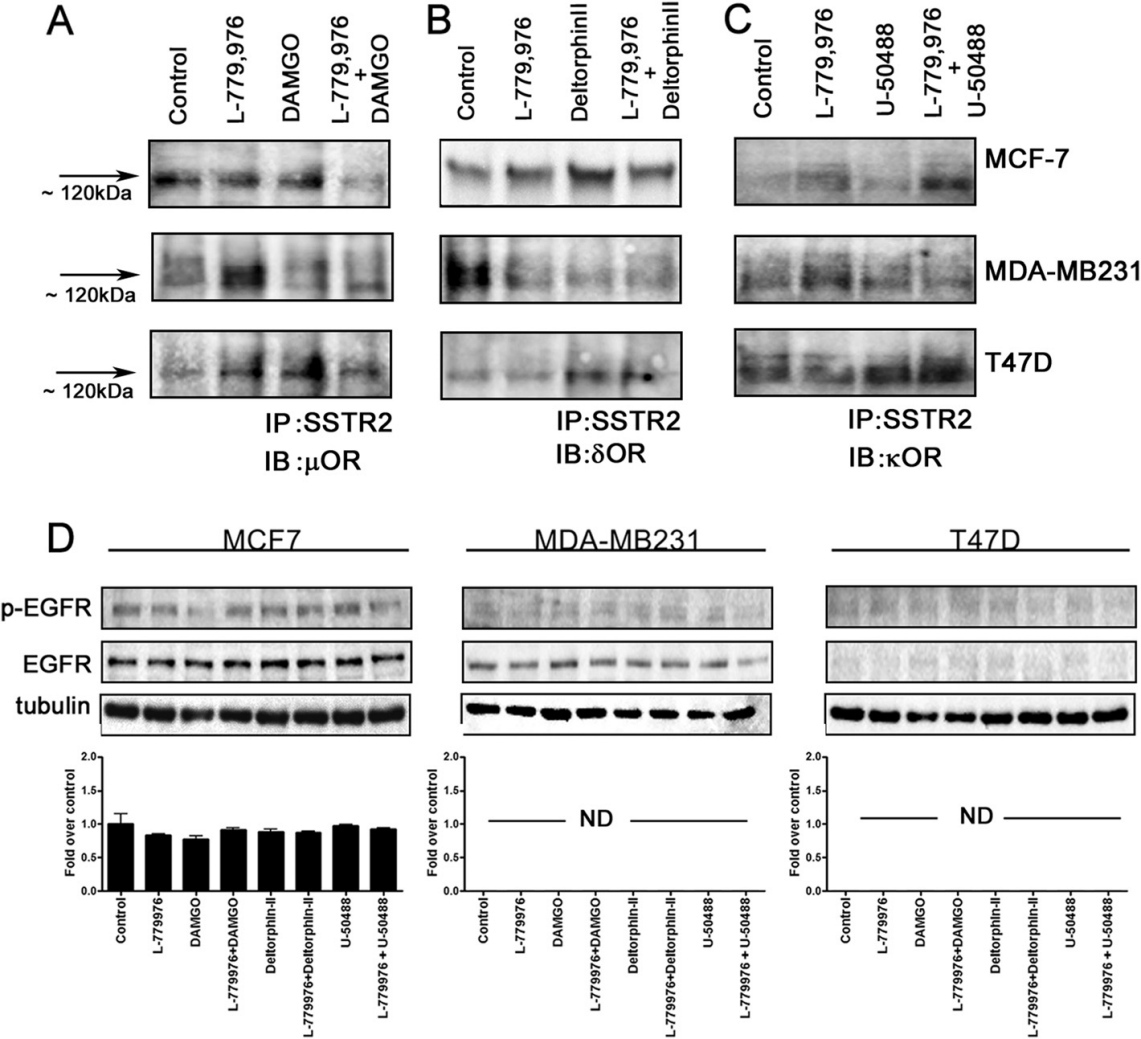
**Agonist dependent complex formation between SSTR2/ORs and inhibition of EGFR phosphorylation.** Co-immunoprecipitation showing the expression of μOR **(A)**, δOR **(B)** and κOR **(C)** in SSTR2 immunoprecipitate obtained from MCF-7, MDA-MB231 and T47D cells following indicated treatment. The agonist-induced heterodimerization between SSTR2 and μOR, δOR or κOR is receptor and cell-specific. **(D)** Western blot showing SSTR2 and ORs mediated inhibition of EGFR phosphorylation in breast cancer cells. MCF-7 cells displayed EGFR phosphorylation comparable to control without any discernible difference upon indicated treatment. Note the lack of EGFR phosphorylation in MDA-MB231 despite basal EGFR expression whereas T47D cells were devoid of EFGR expression and phosphorylation. Tubulin was used as a loading control.

Previous studies using HEK-293 cells have demonstrated that ORs and SSTR2 constituted functional heterodimers with enhanced signaling properties [15]. To decipher the underlying mechanism by which SSTR2/ORs functionally interact in tumor cells expressing these receptors endogenously, SSTR2 immunoprecipitate was processed for the expression of ORs by CO-IP. In MCF-7 cells, SSTR2/μOR exists in a heteromeric complex at the expected molecular size of ~120 kDa in control as well as upon activation of both receptors independently (Figure 6A). However, with combined treatment of L-779,976 + DAMGO, there was significant loss in SSTR2/μOR complex (Figure 6A). In MDA-MB231 cells, complex formation of SSTR2/μOR was weak in basal state. The SSTR2/ μOR complex was enhanced significantly upon activation of SSTR2 and the receptor heterodimerization was lost upon treatments with DAMGO alone or in combination with L-779,876 (Figure 6A). SSTR2 immunoprecipitate prepared from control T47D cells was devoid of μOR expression. However, upon treatments with L-779,876 or DAMGO alone or in combination, SSTR2 immunoprecipitate displayed μOR expression at the expected size of ~120 kDa (Figure 6A).

In comparison to control, the heterodimerization between SSTR2/δOR strengthened upon treatments with specific agonists in MCF-7 cells (Figure 6B). In contrast, MDA-MB231 cells in basal condition displayed strong SSTR2/δOR complex formation and led to the dissociation upon agonists treatment (Figure 6B). In T47D cells, no interaction between SSTR2/δOR in basal as well as upon treatment with L-779,976 was seen. However, upon treatments with Deltorphin-II alone or in combination with L-779,976, cells exhibited significant expression of δOR in SSTR2 immunoprecipitate.

SSTR2 immunoprecipitate prepared from control and U50488HCl treated MCF-7 cells were devoid of κOR expression (Figure 6C). Whereas cells treated with specific agonists for SSTR2 alone or in combination with κOR induced the complex formation between SSTR2/κOR (Figure 6C). In MDA-MB231 cells, activation of SSTR2 alone displayed increased complex formation between SSTR2/κOR however, upon treatments with U50488HCl alone or in combination with L-779,976; the expression of κOR in SSTR2 immunoprecipitate was comparable to control (Figure 6C). Conversely, T47D cells, exhibited comparable expression of κOR in SSTR2 immunoprecipitate in control as well as upon treatment with SSTR2 agonist. However in comparison SSTR2/κOR complex formation increased upon treatment with κOR agonist alone and in combination with SSTR2 agonist (Figure 6C). These results strengthen the concept of ligand, cell dependency in possible heterodimerization between SSTR2 and ORs respectively. The specificity of immunoreactivity was confirmed in absence of primary antibodies and incubation with secondary antibodies alone as previously described [20,21].

### EGFR phosphorylation in breast cancer cells is abolished in presence of synergistic activation of SSTR2 and ORs

The observations that breast tumor progression associated with EGFR over-expression, phosphorylation and homo-and/or heterodimerization suggested a crucial role of EGFR in breast cancer [13,20,21,51]. In order to identify the possible link between EGFR and SSTR2/ORs we determined the effect of SSTR2 and ORs activation on EGFR phosphorylation. In MCF-7 cells, SSTR2 agonist L-779,976 alone or in combination with ORs agonists decreased the phosphorylation of EGFR although not significantly different than the control (Figure 6D). Interestingly, MDA-MB231 and T47D cells displayed no EGFR phosphorylation across all treatments (Figure 6D). These data indicate that SSTR2 and ORs together maintained EGFR phosphorylation comparable to control in MCF-7 cells. The inhibition of EGFR phosphorylation is crucial in breast cancer, however, whether the changes seen in the downstream signaling pathways are in-part due to the lack of EGFR phosphorylation needs further investigation.

## Discussion

In the present study we unfold the effects of activation of SSTR2 and ORs alone or in combination on signaling cascades and cell proliferation in human breast cancer cells including MCF-7, MDA-MB231 and T47D. The activation of SSTR2 and ORs modulate the MAPK pathway and inhibit the cell survival PI3K/AKT signaling molecules and thereby enhancing the expression of tumor suppressor proteins PTEN/p53 in cell and possibly ER dependent manner. These changes in modulation of downstream signaling were corroborated using flow cytometry describing the nature of cell death which showed eminent necrosis indicating the anti-proliferative effect of SSTR2 and ORs in breast cancer cells. In addition, our results describe receptor and agonist dependent expression of ORs in SSTR2 immunoprecipitate suggesting that SSTR2 and ORs might interact as heterodimers. In tumor cells, activation of SSTR2 and ORs inhibit the phosphorylation of EGFR. Although, there is growing understanding for the synergistic effect of many GPCRs in cells transfected with one or more receptor, this is the first comprehensive description of SSTR2 and ORs in tumor cells expressing both receptor subtypes endogenously. Our results demonstrate agonist dependent internalization of SSTR2 and ORs in receptor and cell-specific manner. The activation of SSTR2 and ORs modulate MAPKs (ERK1/2 and p38) and the expression of tumor suppressor proteins, PTEN and p53 resulting in the suppression of PI3K/AKT pathway. We further correlated the effects of SSTR2/ORs mediated signaling with the functional consequences by measuring the apoptosis and necrosis using flow cytometry. The results described here also revealed heterodimerization between SSTR2/OR and inhibition of EGFR phosphorylation. Our findings for the first time highlight the molecular mechanisms for the role of SSTR2 and ORs mediated antagonism of tumorigenic signaling pathways in human breast cancer cells in receptor dependent manner.

The co-expression of given receptors is a pre-requisite for heterodimerization and this was further supported by expression of ORs in SSTR2 immunoprecipitate. Consistent with previous studies in heterologous system, our results showed that SSTR2 exist as pre-formed heterodimers with μ, δ and κ-ORs in breast cancer cells in receptor and cell-specific manner [15]. Moreover, SSTR2 formed heterodimers with δ and κORs in agonist and cell specific manner. Our results strengthen the concept that the activation of one receptor is capable of inducing receptor complex formation while second protomer may either stabilize or dissociate the heteromeric complex.

There is preponderance of evidence suggesting the mechanistic significance of GPCRs expression, trafficking and heterodimerization in modulation of downstream signaling pathways [18,20,21,52]. Studies suggest that the activation of ERKs and p38 in various cancer cells lead to aberrant cell proliferation [53]. Consistent with the existing data, activation of SSTR2 or ORs alone or in combination inhibit ERK1/2 in MCF-7 and T47D cells more than the MDA-MB231 cells [28]. Interestingly, p38 mediated anti-apoptotic effects typically seen in breast cancer are remarkably diminished in MDA-MB231 and T47D cells upon activation of SSTR2 and ORs. Surprisingly, T47D and MCF-7 cells are ER + ve, but the cellular response to SSTR2/OR activation and inhibition of p38 was pronounced in T47D cells. Although such discrepancy in inhibition of p38 may be attributed due to the changes in other isoforms of p38 in addition to the difference in the origin of these cells; MCF-7 (adenocarcinoma) and T47D (ductal carcinoma). However, the intensity of expression levels of ER in these cells cannot be avoided from the discussion and future studies are warranted in this direction. Moreover results described here are consistent with the previous studies showing an enhanced pro-apoptotic effect in MCF-7 cells over-expressing SSTR2 [16].

PI3K/AKT hyper-phosphorylation, uncontrolled breast cancer progression and resistance to hormonal therapy is well established [48,54]. In all three tumor cell lines, the status of phosphorylated PI3K/AKT was not significantly different from the control. Moreover, this blockade of PI3K/AKT specifically in MCF-7 and T47D cells was accompanied by an increased expression of tumor suppressor PTEN supporting the notion that loss of PTEN is due to PI3K/AKT activation [48,49]. Notably, the activation of PI3K/AKT induced ERα expression in MCF-7 cells and spared them from tamoxifen mediated apoptosis [54]. This suggests that enhanced PTEN expression, predominantly in ER + ve MCF-7 and T47D cells as described here may alleviate TAM resistance. The activation of PI3K/AKT has also been shown to down regulate p53 induced apoptosis in breast cancer [55]. Previous reports suggested that in MCF-7 cells with over-expression of ErbB2 significantly activate PI3K and decreased the expression of p53, however, this effect was reversed upon blocking PI3K pathway [56]. Taken into consideration, enhanced expression of p53 and comparable activation of PI3K/AKT to control also highlight novel function of SSTR2/ORs in breast cancer cells.

Given the receptor dependent role, SSTR2 and ORs regulating the MAPK and PI3K/AKT pathways in breast cancer cells is an indication of inhibition of EGFR functions. This speculation is supported by aggressive tumor proliferation in breast cancer displaying over-expression and hyper-phosphorylation of EGFR [24,51,57]. An inverse relationship between EGFR and ER has been established. Moreover, the absence of ER expression in human breast cancer cell lines including MDA-MB231 is associated with higher levels of functional EGFR protein and mRNA [58]. In parallel to previous studies, our data revealed complete blockade of EGFR phosphorylation upon activation of SSTR2 and ORs in MDA-MB231 and T47D cells [59,60]. Conversely, in MCF-7 cells, EGFR phosphorylation was lower than the control suggesting that endogenous EGFR phosphorylation is relatively higher in comparison to other cancer cells. We anticipate that the SSTR2/ORs mediated inhibition of EGFR phosphorylation possibly arrest the downstream MAPK and PI3K/AKT signaling thereby inducing the expression of tumor suppressor PTEN and p53. This supposition is further supported by our recent studies showing the inhibition of EGFR phosphorylation by activation of SSTR1 or SSTR5 leading to the pronounced inhibition of cell proliferating signals in HEK-293 cells [20,21]. Moreover, the physiological response of tumor cells upon activation of SSTR2/ORs parallel the changes seen in downstream signaling. FACs analysis revealed higher necrotic cell population (>25%) in MCF-7 and T47D cells than MDA-MB231 cells. The early apoptosis increased upon specific treatment, however, was limited to <5%. Consistent with previous studies, our observations suggest a much pronounced cytotoxic role of SSTR2 and ORs in breast cancer cells as described previously [16]. These observations warrant concentration and time dependent effect of SSTR2/OR agonist on apoptosis and necrosis and further studies are in progress in this direction.

Targeting EGFR hyperactivity and PI3K/AKT pathways have been the therapeutic approach for the treatment of breast cancer. SSTR2 agonists have been clinically used for the treatment of acromegaly and pancreatic tumors, whereas ORs are emerging new members of GPCR family for their anti-proliferative role in various tumors. Therefore, targeting SSTR2 and specific ORs could possibly be a better therapeutic approach in breast cancer treatment. In addition to the anti-proliferative role, the analgesic role of ORs in the treatment of breast cancer cannot be neglected since patients undergoing chemotherapy are known to experience pain. Previously, SST analogs have been proven to be effective in pain relief in cases where opioids therapy failed [61]. It would be worth investigating the dual role of ORs as a tumor suppressor and analgesic agent in the treatment of breast cancer in synergism with SSTR subtypes. Our observations uncovered the new role of SSTR2 and ORs in combination in regulating the key tumor promoting signals in breast cancer cells. To determine whether such effect is partly due to direct functional interaction between these receptors further studies are in progress in this direction. Taken together, results presented in this study in part may be due the presence and expression intensity of ER. Furthermore, additional studies are essential to support whether knocking down ER in MCF-7 and T47D and transient expression of ER in MDA-MB231 cells display comparable changes. In conclusion, this is the first comprehensive study unveiling the molecular mechanisms of SSTR2/ORs mediated anti-proliferative signaling with novel therapeutic implications in breast cancer treatment.

## Competing interests

The authors declare that they have no competing interests.

## Authors’ contributions

GK and UK conceived, designed and wrote the manuscript. GK performed experiments and analyzed the data. PSR performed microscopy and YL performed western blot. All authors read and approved the manuscript.
